# Meta-Analysis of Genome-Wide Association Studies Identifies Novel Functional CpG-SNPs Associated with Bone Mineral Density at Lumbar Spine

**DOI:** 10.1155/2018/6407257

**Published:** 2018-08-07

**Authors:** Chuan Qiu, Hui Shen, Xiaoying Fu, Chao Xu, Hongwen Deng

**Affiliations:** ^1^Department of Global Biostatistics and Data Science, Center for Bioinformatics and Genomics, School of Public Health and Tropical Medicine, New Orleans 70112, USA; ^2^School of Basic Medical Science, Central South University, Changsha 410013, China

## Abstract

Osteoporosis is a serious public health issue, which is mostly characterized by low bone mineral density (BMD). To search for additional genetic susceptibility loci underlying BMD variation, an effective strategy is to focus on testing of specific variants with high potential of functional effects. Single nucleotide polymorphisms (SNPs) that introduce or disrupt CpG dinucleotides (CpG-SNPs) may alter DNA methylation levels and thus represent strong candidate functional variants. Here, we performed a targeted GWAS for 63,627 potential functional CpG-SNPs that may affect DNA methylation in bone-related cells, in five independent cohorts (*n* = 5905). By meta-analysis, 9 CpG-SNPs achieved a genome-wide significance level (*p* < 7.86 × 10^−7^) for association with lumbar spine BMD and additional 15 CpG-SNPs showed suggestive significant (*p* < 5.00 × 10^−5^) association, of which 2 novel SNPs *rs7231498 (NFATC1)* and *rs7455028 (ESR1)* also reached a genome-wide significance level in the joint analysis. Several identified CpG-SNPs were mapped to genes that have not been reported for association with BMD in previous GWAS, such as *NEK3* and *NFATC1* genes, highlighting the enhanced power of targeted association analysis for identification of novel associations that were missed by traditional GWAS. Interestingly, several genomic regions, such as *NEK3* and *LRP5* regions, contained multiple significant/suggestive CpG-SNPs for lumbar spine BMD, suggesting that multiple neighboring CpG-SNPs may synergistically mediate the DNA methylation level and gene expression pattern of target genes. Furthermore, functional annotation analyses suggested a strong regulatory potential of the identified BMD-associated CpG-SNPs and a significant enrichment in biological processes associated with protein localization and protein signal transduction. Our results provided novel insights into the genetic basis of BMD variation and highlighted the close connections between genetic and epigenetic mechanisms of complex disease.

## 1. Introduction

Osteoporosis is a complex disease mainly characterized by low bone mineral density (BMD) and microarchitectural deterioration of bone tissue, which results in an increased risk of bone fragility and susceptibility to fracture [1]. It is an increasingly serious public health issue in the aging population; the prevalence of osteoporosis at lumbar spine in the elderly is over 20% in the United States [2]. Genetic studies have demonstrated that BMD is under strong genetic control, with heritability ranging between 50 and 85% [3, 4]. Genome-wide association studies (GWAS) and meta-analyses of these studies have successfully identified over 250 genetic loci associated with BMDs at different skeletal sites [5–11]. However, these loci explained approximately 12% of BMD variation [11] and the specific functional variants at these loci were generally unknown. To search for additional genetic loci and to enhance our understanding of the biological mechanisms underlying BMD variation, one effective strategy is to focus on testing of specific variants with high potential of functional effects, such as exonic/nonsynonymous variants [5, 12] or variants that may potentially affect regulatory factors [13–16]. Such strategy can alleviate the multiple testing problem of the conventional GWAS approach and consequently enhance the power to identify novel functional variants associated with the phenotype of interest. In addition, because the hypothesis testing is based on SNPs with potential functions, false positive findings may be minimized due to that the information on prior functional evidence is used.

DNA methylation is an essential epigenetic mechanism for the regulation of transcription. It has profound impacts on chromatin structure, genomic imprinting, embryonic development, X-chromosome inactivation, and the pathogenesis of several human genetic disorders [17]. Although epigenetic regulation by DNA methylation is generally thought to be transcriptionally repressed in gene promoters and transcriptionally activated when occurring in gene bodies [18, 19], recent studies suggested a much more complex relationship between DNA methylation and the gene expression pattern. Both positive and negative associations between DNA methylation and gene expression have been revealed across all genomic regions of a gene, and DNA methylation can also modulate alternative RNA splicing via regulation of the RNA Pol II elongation rate [20–24], demonstrating that DNA methylation can have diverse, chromatin cell type- and context-dependent regulatory effects of transcription.

Single nucleotide polymorphisms (SNPs) may introduce or disrupt cytosine-phosphate-guanine dinucleotides (CpG sites), the major substrate for methyl transfer reactions, and therefore dramatically alter the methylation status at the affected loci [25]. These so-called CpG-SNPs have been suggested as an important mechanism through which genetic variants can affect gene function via epigenetics [25, 26]. Shoemaker et al. performed genome-wide allele-specific methylation analysis in 16 human cell lines and found that a significant proportion (38–88%) of allele-specific methylation regions relied on the presence of CpG-SNP variations [27]. Similarly, Zhi et al. conducted genome-wide correlation analysis between genetic variants and DNA methylation levels in human blood CD4+ T cells and found that over 80% of CpG-SNPs were local methylation quantitative trait loci (*cis*-meQTLs) and CpG-SNPs accounted for over 2/3 of the strongest meQTL signals [28]. The effect of CpG-SNPs often extended beyond the directly affected CpG sites to surrounding regions, likely via correlated proximal methylation patterns and genetic linkage disequilibrium (LD) [25, 28, 29]. These evidences strongly suggested that CpG-SNPs are a crucial type of *cis*-regulatory polymorphic variants connecting genetic variation to the individual variability in epigenome. By focusing on CpG-SNPs in selected candidate genes, several studies have identified significant associations between CpG-SNPs with human complex disorders, such as breast cancer [30], type 2 diabetes [29], alcohol dependence [31], and suicide attempt in schizophrenia [32], implying that focusing on CpG-SNPs is an efficient strategy to identify novel functional variants underlying human complex disorders/traits.

In this study, we performed a targeted GWAS analysis for BMD on CpG-SNPs. As DNA methylation profiles are often cell-type specific [33], we further narrowed down to CpG-SNPs that are also meQTLs in an osteoclast-lineage cell, specifically, human peripheral blood monocytes (PBMs). PBMs can act as precursors of osteoclasts, produce cytokines important for osteoclast differentiation and function, serve as a major target cell of sex hormones for bone metabolism [34–38], and have been demonstrated as an excellent cell model for studying osteoporosis-related gene/protein expression patterns and their regulatory mechanisms [39–50]. Therefore, our targeted potential functional CpG-SNPs represent prominent candidates that can regulate BMD variation by affecting gene activity via epigenetic mechanisms in bone-related cells.

## 2. Materials and Methods

### 2.1. Study Cohorts

The discovery dataset incorporated a total of 5905 subjects from five GWAS, of which three studies were “in-house” studies: (1) Omaha Osteoporosis Study (Caucasian ancestry, *n* = 987), (2) Kansas City Osteoporosis Study (Caucasian ancestry, *n* = 2250), and (3) China Osteoporosis Study (Han Chinese ancestry, *n* = 1547), and two studies were “external” studies obtained from the Database on Genotypes and Phenotypes (dbGaP): (1) Women's Health Initiative Observational Study African-American Substudy (African ancestry, *n* = 712) and (2) Women's Health Initiative Observational Study Hispanic Substudy (Hispanic ancestry, *n* = 409). The basic characteristics of the five study cohorts were shown in Supplementary Table 1. All studies were reviewed and approved from respective institutional review boards, and each eligible participant provided written informed consent for enrolment. The replication dataset included the summary statistics for the association of approximately 10 million SNPs with BMD by the Genetic Factors for Osteoporosis Consortium (GEFOS) [5, 8]. To our knowledge, it is the largest GWAS meta-analysis dataset for BMD association to date in the bone field [5, 8].

### 2.2. Selecting Potential Functional CpG-SNPs

The CpG-SNPs that are potentially functional in PBMs were selected according to the following steps:

1. We identified CpG-SNPs in the human genome by interrogating the extensive catalog of common and rare genetic variants from the 1000 Genomes reference panel [51] and our in-house whole-genome high-coverage deep resequencing study [52]. A SNP was defined as a CpG-SNP if it introduces or disrupts a CpG site. A total of 3,363,517 CpG-SNPs was identified throughout the human genome.
2. We retrieved 39,859 PBM meQTLs at a stringent significance threshold (FDR < 0.001) from the previous study that assessed the association of over 7 million SNPs with methylome of PBMs in 200 unrelated individuals [53]. We then used SNiPA [54] to identify proxy SNPs in strong LD with retrieved PBM meQTLs. The search was depended on genotype information from the 1000 Genomes Project with the European samples [51]. The inclusion criteria for proxy SNPs were set as a pairwise *r* ^2^ threshold > 0.9 and a distance limit of 10 kb from the query meQTL. A total of 175,710 potential PBM DNA methylation-associated SNPs (reported meQTLs and proxy of meQTLs) were identified.
3. By finding common SNPs between the CpG-SNPs and PBM DNA methylation-associated SNPs, we identified a total of 68,041 CpG-SNPs that are potentially functional in PBMs.

### 2.3. BMD Measurements

The lumbar spine BMD was determined by either the Hologic Inc. (Bedford, MA, USA) or GE Lunar Corp. (Madison, WI, USA) dual-energy X-ray absorptiometry (DXA) scanner following the respective manufacturer's scan protocols. For each GWAS, multiple potential covariates such as scanner ID, sex, height, weight, age, and age^2^ were screened using a forward stepwise linear regression. The significant covariates were used to adjust for raw BMD measurements. Correction of potential population stratification was performed with principal component analysis (PCA), and the top five PCs (i.e., PC1–PC5) were also included as covariates. Residual scores of adjusted phenotypes were normalized by inverse quantile of the standard normal distribution, which was analyzed subsequently.

### 2.4. Genotyping and Quality Control

For each GWAS, genome-wide genotyping was performed by either Affymetrix Inc. (Santa Clara, CA, USA) or Illumina Inc. (San Diego, CA, USA) high-density SNP genotyping platforms following respective manufacturer's assay protocols. Quality control was implemented by PLINK (http://pngu.mgh.harvard.edu/~purcell/plink/) with the following criteria: individual missingness < 5%, SNP with successful call rate > 95%, and Hardy-Weinberg equilibrium *p* value > 1.0 × 10^−5^. PCs derived from genome-wide genotyping analysis were used to monitor the population outliers.

### 2.5. Genotype Imputation

To allow for the merging of datasets from different types of genotyping platform to obtain higher depth of genome coverage, we performed extensive genotype imputation analysis. Generally, haplotype inference of each GWA study was initially phased by a Markov Chain Haplotyping algorithm (MACH) [55] and Minimac [56] was then used to impute genotypes at untyped variants based on haplotype data from the 1000 Genomes reference panel [51]. For each GWA study, the haplotype reference panel of relevant population was used to impute genotypes at untyped variants. SNPs with *imputation quality score* (*r* ^2^) > 0.3 and minor allele frequency (MAF) > 0.05 in no less than 2 studies were retained in the subsequent analyses. Imputation with the 1000 Genomes Project reference panels generated genotype data for more than 11.2 million SNPs. Among the 68,041 potential functional CpG-SNPs, 63,627 CpG-SNPs had qualified genotype data (genotyped + imputed) and thus were tested in the following GWAS meta-analyses.

### 2.6. Association Tests and Meta-Analyses

For each GWAS, we test the association between directly typed/imputed SNPs and lumbar spine BMD using an additive genetic model. The association of unrelated subjects in each GWAS was tested by fitting a linear regression model with MACH2QTL [55] in which allele dosage was considered as a phenotype predictor. The genomic inflation factor (*λ*_GC_) [57] was also estimated for each individual GWAS. We performed meta-analysis using software METAL [58] which based on weights proportional to the square root of the number of subjects in each sample, and between-study heterogeneity was estimated by *Cochran*'s *Q* statistic and *I* ^2^. Genome-wide significance threshold was defined as a *p* value < 7.86 × 10^−7^ (Bonferroni correction for testing 63,627 selected CpG-SNPs).

### 2.7. Function Annotation of the CpG-SNPs

CpG-SNPs were annotated with SNPnexus [59] based on reference genome GRCh37 and assigned to candidate genes (±2 kb upstream and downstream). In order to test the potential functional importance of the identified CpG-SNPs, we applied HaploReg [60] to annotate selected CpG-SNPs to enhancer histone marks (H3K4me1/H3K27ac) across diverse tissue/cell types from the Roadmap Epigenomics Projects and test the effect of SNPs on changing the regulatory motifs and the effect of SNPs on the regulation of gene expression of target genes. We employed the software GOEAST [61] to identify significant gene ontology terms among genes associated with identified novel functional CpG-SNPs in lumbar spine.

## 3. Results

In this study, we identified 68,041 potential functional CpG-SNPs that may both affect DNA methylation by introducing or disrupting CpG sites and influence DNA methylation levels in human PBMs. Interestingly, although over 50% of these potential functional CpG-SNPs were mapped to introns, we observed a significant enrichment of potential functional CpG-SNPs in 5′/3′-UTR regions (fold change > 2) and underrepresentation in intergenic regions (Supplementary Figure 1), when comparing to the overall profile of CpG-SNPs in the human genome.

**Figure 1:**
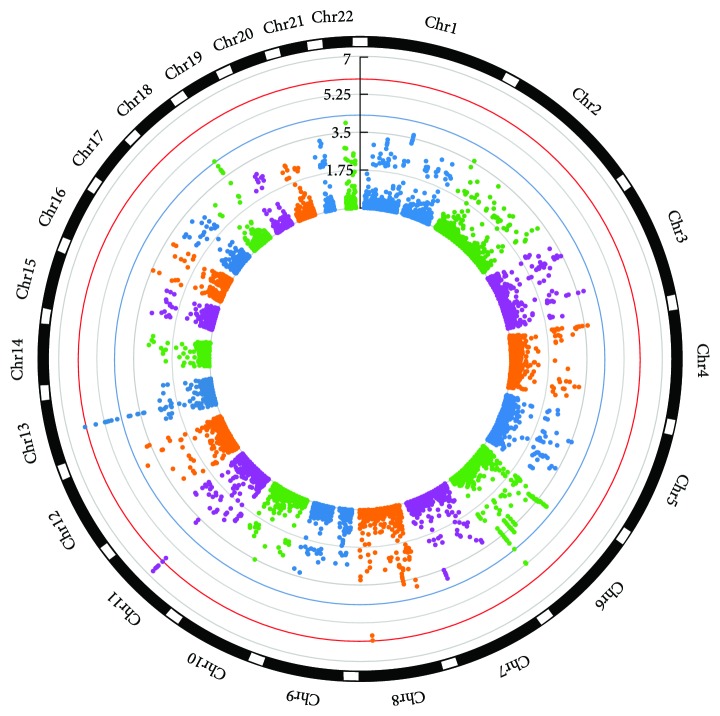
Circular Manhattan plot picturing the −log_10_ (*p* values) of meta-analysis results for lumbar spine BMD. CpG-SNPs were plotted according to the chromosomal location. The blue and red circular lines indicate the threshold for suggestive significant (*p* value = 5.00 × 10^−5^) and significant SNPs (*p* value = 7.86 × 10^−7^), respectively.

**Figure 2:**
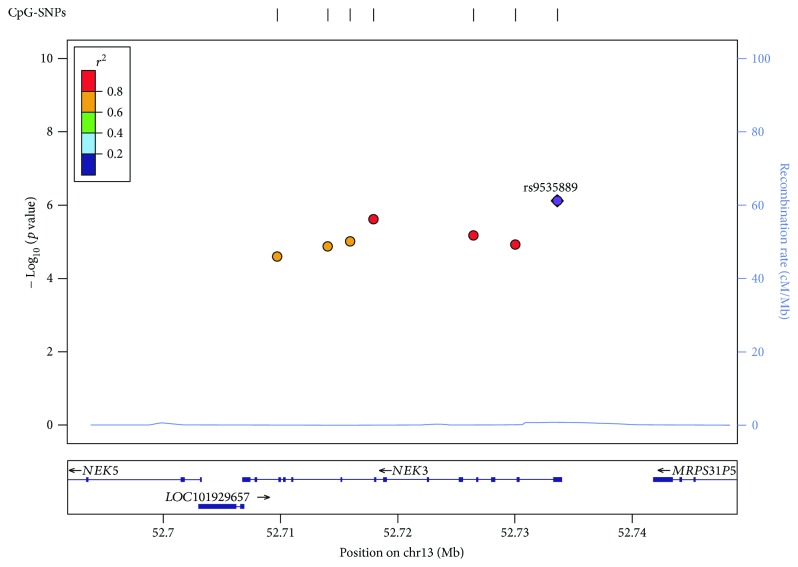
A regional association plots of significant/suggestive CpG-SNPs at *NEK3* regions. Genes and expressed sequence tags (ESTs) within the region are shown in the lower panel, and the unbroken blue line indicates the recombination rate within the region. Each filled circle represents the *p* value for one SNP in the meta-analysis, with the top SNP *rs9535889* shown in purple and SNPs in the region colored depending on their degree of LD (*r* ^2^) with *rs9535889*. LD was estimated by LocusZoom [80] on the basis of CEU (Utah residents of Northern and Western European ancestry) HapMap haplotype data.

We successfully obtained genotype data for 63,627 potential functional CpG-SNPs and carried out targeted association studies in five independent GWAS cohorts with a total of 5905 subjects. The estimates of genomic inflation factor *λ*_GC_ ranged from 0.97 to 1.02 in individual GWAS. By performing meta-analysis combining the five GWAS datasets, we identified 9 CpG-SNPs that were significantly associated with lumbar spine BMD at a genome-wide significance level (*α* = 7.86 × 10^−7^), including 5 novel SNPs *rs689179* (*p* value = 2.68 × 10^−7^), *rs576118* (*p* value = 2.70 × 10^−7^), *rs471966* (*p* value = 3.29 × 10^−7^), *rs640569* (*p* value = 4.04 × 10^−7^), and *rs667126* (*p* value = 7.80 × 10^−7^) in *LRP5* gene and one SNP *rs9535889* in novel gene *NEK3* (*p* value = 7.55 × 10^−7^). We also confirmed 3 previously reported loci (*rs525592*, *rs1784235*, and *rs497261*) in *LRP5* gene (Figure 1 and Table 1). In addition, 15 CpG-SNPs achieved a suggestive significance level (*α* = 5.00 × 10^−5^) for association with lumbar spine BMD (Figure 1 and Table 1). We then performed in silico replication for the identified 24 significant/suggestive CpG-SNPs in the GEFOS cohort [5, 8] and successfully replicated (*p* value < 0.05) 14 CpG-SNPs (Table 1). Subsequently, a joint analysis of both the discovery and replication studies identified 2 additional novel CpG-SNPs associated with lumbar spine BMD at a genome-wide significance level (Table 1) including the SNP *rs7455028* (*p* value = 1.18 × 10^−7^) in *ESR1* gene and the SNP *rs7231498* (*p* value = 7.18 × 10^−7^) in *NFATC1* gene. A number of the significant/suggestive CpG-SNPs were clustered into the genomic regions encompassing *NEK3* and *LRP5* genes (Figure 2 and Supplementary Figure 2). These clustered CpG-SNPs are in high LD and therefore, may represent the same functional loci that synergistically mediate the DNA methylation and/or gene expression of their target genes.

To further explore the potential functional significance of the identified significant/suggestive CpG-SNPs, we annotated these CpG-SNPs to various chromatin states and other possible regulatory elements with data from Roadmap Epigenomics and GTEx projects through the HaploReg program [60]. The chromatin state and histone modification data suggested the evidence of regulatory potential in the identified CpG-SNPs. 20 CpG-SNPs altered the regulatory motif, along with 14 CpG-SNPs involved enhancer histone markers. Notably, the novel SNPs *rs9535889*, *rs9526841*, and *rs2408611* in *NEK3* gene were all located in regions with strong transcription and enhancer activities in PBMs as well as various other tissues and cell types (Table 2), highlighting strong regulatory potential of these CpG-SNPs. In addition, many identified CpG-SNPs may affect binding of various transcription factors and have numerous reported eQTL evidences in various tissue/cell types (Table 2). We also conducted gene ontology analysis for the genes related to the identified CpG-SNPs and revealed significant enrichment of biological processes which are closely associated to protein localization and protein signal transduction (Table 3), such as protein localization to plasma membrane/cell periphery and regulation of Ras/Rho protein signal transduction gene ontology terms.

## 4. Discussion

Our study represents the first targeted GWAS testing CpG-SNPs that are potentially functional in bone-related cells for association with BMD variation. As epigenomic and transcriptomic profiles are often tissue-/cell-type specific, we speculated that only a subset of CpG-SNPs in the human genome will have functional impact on DNA methylation levels in specific tissues/cells. Therefore, it is necessary to select out those CpG-SNPs that are potentially functional in disease-/trait-related tissues/cells when performing CpG-SNP-focused association studies. One reasonable and efficient filtering strategy is to leverage the enormous available meQTL data in diverse tissues/cells. Unfortunately, meQTL data in skeletal cells were scarce; therefore, we used meQTL data from PBMs to select out 68,041 candidate CpG-SNPs that may be functional in regulating bone mass, considering the direct and close connections between PBMs and bone metabolism. These potential functional CpG-SNPs were enriched in 5′/3′-UTR regions but underrepresented in intergenic regions (Supplementary Figure 1). This result is largely in line with the recent findings in tissue-/cell-type specific DNA methylation profiles, suggesting that methylation-mediated regulatory effects often occur beyond the promoter areas [18, 62].

By using the data from five independent GWAS cohorts and the summary statistics from the GEFOS study, we identified significant/suggestive associations for 24 CpG-SNPs with lumbar spine BMD. These BMD-associated CpG-SNPs were mapped to six genes; some of which have not been reported for association with BMD in previous GWAS, such as *NEK3* and *NFATC1* genes. Our finding highlighted the enhanced power of targeted association analysis for identification of novel associations that were missed by traditional GWAS. Interestingly, several genomic regions, such as *LRP5* and *NEK3* regions, contained multiple significant/suggestive CpG-SNPs, suggesting that multiple neighboring CpG-SNPs may synergistically mediate the DNA methylation and gene expression of the target genes. This is consistent with the fact that methylation signals among neighboring CpG sites are often strongly correlated and regulatory elements that were mediated by methylation usually extend across various genomic regions [63]. *LRP5* gene encodes a transmembrane protein which acts as a receptor for low-density lipoprotein. This transmembrane receptor initializes the process of receptor-mediated endocytosis by binding and internalizing their corresponding ligands [64]. It is well known for the critical role in bone homeostasis and several skeletal disorders [65]. Several common genetic variants of *LRP5* gene have been demonstrated as potential risk factors in osteoporosis and fracture by previous GWAS [66, 67]. For example, gain of functional variations in *LRP5* gene leads to extremely high BMD [64] and loss of functional variations in *LRP5* gene results in osteoporosis-pseudoglioma syndrome [68]. Interestingly, the recent study showed that the differentiation of monocytes can be negatively regulated by *LRP5* gene through abrogation of the Wnt pathway which has an essential role in bone remodeling in both physiological and pathological conditions [69]. The other interesting gene is the *NEK3*, which also contained several significant/suggestive CpG-SNPs and enriched with strong transcription and enhancer histone modification marks in PBMs and a variety of other tissue/cell types. *NEK3* gene encodes a member of the NimA-related serine/threonine kinases [70]. These kinases have been implicated as the significant regulators of cell migration [71] and also regulate microtubule acetylation in neurons [72]. Although most of these CpG-SNPs were annotated to introns of the *NEK3* gene, the eQTL data from GTEx project suggested that these CpG-SNPs were strongly associated with the expression of *NEK3* gene in diverse tissues. Notably, the previous study [73] that assessed the association of over 675,000 SNPs with transcriptome of PBMs in 1490 unrelated individuals showed that SNP *rs2408611* in *NEK3* gene has a strong *cis*-eQTL effect in PBM. This evidence may support that CpG-SNP-mediated epigenomic alterations may be an important mechanism underlying the association between *NEK3* and BMD variation. However, its function in other tissues, including bone, remains largely uncharacterized. Another interesting gene is *NFATC1*. This gene encodes a transcription factor involved in T cell maturation. Importantly, *NFATC1* can also regulate activity of a number of osteoclast-specific enzymes and/or other molecules, such as osteoclast-associated receptor, TRAP, calcitonin receptor, and cathepsin K through cooperation with MITF and c-Fos [74–77]. The important role of this gene in differentiation of osteoclast has been well established by several studies performed on genetically modified mutant mice [78, 79]. For example, Winslow et al. [78] identified that the transgenic mice generated by crossing *NFATC1*-knockout mice with mice that express Tie2 promoter-driven *NFATC1* exhibit an osteopetrotic bone phenotype, which may result from a severe defect in the osteoclastogenesis process. Therefore, understanding the molecular basis underlying the functional regulation of *NFATC1* in osteoclasts may provide novel therapeutic strategies for bone diseases.

Several potential limitations of this study should be concerned and addressed in the future. First, the selection of an appropriate cell model is crucial. Due to the limited meQTL studies in the bone cell models, here, we focused on CpG-SNPs that are also meQTLs in an osteoclast-lineage cell, specifically, human PBMs. Although PBMs act as precursors of osteoclasts and act as the major target cells of sex hormones for bone metabolism, the ideal model cells for the osteoporosis study are bone cells, such as osteoblast, osteoclast, and osteocyte. Second, the results of functional annotation exclusively depend on computationally predicted regulation features and further experimental validation should be conducted to confirm the biological significance of these potential functional CpG-SNPs.

In summary, we performed a targeted GWAS analysis for potential functional CpG-SNPs and identified 2 novel BMD-associated genes, *NEK3* and *NFATC1*. Our results highlighted the power of targeted analysis of potential functional variants for the identification of novel disease susceptibility loci that have been missed by a conventional GWAS approach. More importantly, our findings suggested that CpG-SNP-mediated DNA methylation changes may be a crucial biological mechanism to be considered in the interpretation of associations between common genetic variants, epigenetic process, and phenotypes of human diseases.

## Figures and Tables

**Table 1:** Significant/suggestive CpG-SNPs for lumbar spine BMD.

CpG-SNP	Chr	Position	Alleles	Nearest gene	Feature	Meta *p* value	GEFOS *p* value	Joint *p* value
*rs2941741*	6	152008982	G/A	*ESR1*	Intronic	6.50*E* − 06	1.21*E* − 08	**2.45*E* − 12**
*rs3020333*	6	152010254	A/G	*ESR1*	Intronic, 5′ upstream	7.17*E* − 06	1.57*E* − 09	**3.73*E* − 13**
**rs7455028**	6	152034386	C/T	*ESR1*	Intronic	4.57*E* − 05	0.00013	**1.18*E* − 07**
*rs13254554*	8	120010805	T/C	*COLEC10/TNFRSF11B*	Intronic	8.99*E* − 07	1.10*E* − 19	**5.79*E* − 24**
*rs2220189*	8	120007708	C/G	*COLEC10/TNFRSF11B*	Intronic	1.53*E* − 06	4.25*E* − 20	**3.84*E* − 24**
*rs525592*	11	68195104	C/T	*LRP5*	Intronic	**1.86*E* − 07**	8.69*E* − 11	**6.41*E* − 16**
**rs689179**	11	68179166	A/G	*LRP5*	Intronic	**2.68*E* − 07**	NA	NA
**rs576118**	11	68177708	G/A	*LRP5*	Intronic	**2.70*E* − 07**	3.81*E* − 06^∗^	**2.95*E* − 11 ** ^∗^
**rs471966**	11	68173861	C/T	*LRP5*	Intronic	**3.29*E* − 07**	NA	NA
*rs1784235*	11	68185500	C/T	*LRP5*	Intronic	**3.92*E* − 07**	2.95*E* − 08	**3.82*E* − 13**
**rs640569**	11	68184820	A/G	*LRP5*	Intronic	**4.04*E* − 07**	2.17*E* − 08	**2.92*E* − 13**
**rs667126**	11	68177728	C/T	*LRP5*	Intronic	**7.80*E* − 07**	NA	NA
*rs497261*	11	68192244	T/C	*LRP5*	Intronic	**7.83*E* − 07**	1.86*E* − 11	**5.79*E* − 16**
*rs314751*	11	68179560	C/T	*LRP5*	Intronic	1.31*E* − 06	1.20*E* − 11	**6.24*E* − 16**
*rs23691*	11	68178668	G/A	*LRP5*	Intronic	1.33*E* − 06	1.17*E* − 11	**6.18*E* − 16**
*rs531163*	11	68194496	A/G	*LRP5*	Intronic	1.35*E* − 06	9.05*E* − 11	**4.60*E* − 15**
**rs9535889**	13	52733634	C/G	NEK3	Intronic, 5′ upstream, 5′ UTR	**7.55*E* − 07**	0.469098	5.61*E* − 06
rs3783242	13	52717950	C/T	NEK3	Intronic, 3′ downstream	2.41*E* − 06	NA	NA
rs9526841	13	52726476	A/G	NEK3	Intronic, 3′ downstream	6.64*E* − 06	0.325285	3.03*E* − 05
rs2897976	13	52715944	G/A	NEK3	Intronic	9.71*E* − 06	0.471396	6.08*E* − 05
rs9526843	13	52730056	C/T	NEK3	Intronic	1.17*E* − 05	NA	NA
rs2408609	13	52714043	C/T	NEK3	Intronic	1.32*E* − 05	0.546887	9.26*E* − 05
rs2408611	13	52709742	G/A	NEK3	Intronic, 5′ upstream	2.50*E* − 05	NA	NA
**rs7231498**	18	77189387	A/G	NFATC1	Intronic	4.22*E* − 05	0.000944	**7.18*E* − 07**

Note: CpG-SNPs reached a genome-wide significance level (*p* value ≤ 7.86 × 10^−7^) in discovery meta-analysis and/or joint analysis of discovery, and replication studies are marked in bold. Gene/CpG-SNP reported in previous GWAS for BMD is marked in italics. NA: SNPs were not available in the GEFOS 2015 data release. ^∗^This result was based on the GEFOS 2012 data release because this SNP is not available in the 2015 release.

**Table 2:** Functional annotation of significant/suggestive CpG-SNPs.

CpG-SNPs	Nearest gene	Chromatin state in PBMs^1^	Tissues/cells with enhancer histone marks (H3K4me1/H3K27ac)	Motifs changed	eQTL hits
rs2941741	ESR1	Quiescent/low			
rs3020333	ESR1	Quiescent/low	Liver	Pou2f2	
rs7455028	ESR1	Quiescent/low		5 altered motifs	
rs13254554	COLEC10/TNFRSF11B	Quiescent/low		TCF12, p53	3 hits
rs2220189	COLEC10/TNFRSF11B	Quiescent/low	7 tissues		2 hits
rs525592	LRP5	Quiescent/low		4 altered motifs	2 hits
rs689179	LRP5	Quiescent/low	6 tissues	5 altered motifs	1 hit
rs576118	LRP5	Quiescent/low	IPSC, muscle, heart	TAL1	1 hit
rs471966	LRP5	Quiescent/low	8 tissues	8 altered motifs	2 hits
rs1784235	LRP5	Quiescent/low	Blood	AP-2, ELF1, Rad21	1 hit
rs640569	LRP5	Quiescent/low	Blood	Irf, Pax-4, Pou2f2	1 hit
rs667126	LRP5	Quiescent/low	IPSC, muscle, heart	9 altered motifs	2 hits
rs497261	LRP5	Quiescent/low	Muscle	Pax-5, Smad	5 hits
rs314751	LRP5	Quiescent/low	6 tissues	4 altered motifs	4 hits
rs23691	LRP5	Quiescent/low	6 tissues	5 altered motifs	4 hits
rs531163	LRP5	Quiescent/low		7 altered motifs	3 hits
rs9535889	NEK3	Active TSS	24 tissues^2^	Rad21, SP1, TATA	72 hits
rs3783242	NEK3	Quiescent/low		CDP, Pou2f2	72 hits
rs9526841	NEK3	Strong transcription		HIF1, RFX5, TCF11::MafG	62 hits
rs2897976	NEK3	Quiescent/low			79 hits
rs9526843	NEK3	Quiescent/low	Intestine		73 hits
rs2408609	NEK3	Quiescent/low		6 altered motifs	78 hits
rs2408611	NEK3	Strong transcription		4 altered motifs	80 hits
rs7231498	NFATC1	Weak transcription	Blood	7 altered motifs	

Note: ^1^Chromatin state information was retrieved using a 15-state model from the Roadmap Epigenomics Project based on the 5 core histone marks. ^2^Tissues/cells with promoter histone marks (H3K4me3/H3K9ac). Abbreviation: IPSC: induced pluripotent stem cells.

**Table 3:** The top ten most significant GO terms enriched for BMD-associated CpG-SNPs.

GOID	Term	Log odds ratio	*p* value
GO:0072659	Protein localization to plasma membrane	5.42	4.06*E* − 16
GO:1990778	Protein localization to cell periphery	5.42	4.06*E* − 16
GO:0007009	Plasma membrane organization	4.96	3.08*E* − 14
GO:0035023	Regulation of Rho protein signal transduction	4.57	1.21*E* − 12
GO:0046578	Regulation of Ras protein signal transduction	4.01	1.96*E* − 10
GO:0072657	Protein localization to membrane	3.94	3.45*E* − 10
GO:0010256	Endomembrane system organization	3.93	3.75*E* − 10
GO:0000904	Cell morphogenesis involved in differentiation	2.92	4.50*E* − 08
GO:0051056	Regulation of small GTPase-mediated signal transduction	3.29	7.92*E* − 08
GO:0008295	Spermidine biosynthetic process	7.99	2.55*E* − 07

Note: GO enrichment analysis was performed in candidate genes annotated to BMD-associated CpG-SNPs (*p* value < 1.0 × 10^−4^).

## Data Availability

The chromatin data used to support the findings of this study have been deposited in the Roadmap Epigenomics Project repository. The genotype data used to support the findings of this study are available from the corresponding author upon request.

## Supplementary Materials

Supplementary Materials Supplementary Table 1: basic characteristics of the studied samples. Supplementary Figure 1: distribution of CpG-SNPs in distinct genomic features. Supplementary Figure 2: a regional association plots of significant/suggestive CpG-SNPs at LRP5 regions.
