# Supplementary material for: Meta-Analysis of Genome-Wide Association Studies Identifies Novel Functional CpG-SNPs Associated with Bone Mineral Density at Lumbar Spine
Source: Int J Genomics. 2018 Aug 7;2018:6407257. doi: 10.1155/2018/6407257 (PMC6109501; doi:10.1155/2018/6407257)
Supplement: Supplementary Materials — Supplementary Table 1: basic characteristics of the studied samples. Supplementary Figure 1: distribution of CpG-SNPs in distinct genomic features. Supplementary Figure 2: a regional association plots of significant/suggestive CpG-SNPs at LRP5 regions. [file 6407257.f1.docx]

**Supplementary Materials**

**Supplementary Table 1. Basic Characteristics of the Studied Samples**

| Sample | Sample Size | Population | Female (%) | Age | Height | Weight | LS BMD | Bone densitometer |
| --- | --- | --- | --- | --- | --- | --- | --- | --- |
|  |  |  |  | (yrs) | (m) | (kg) | (g/cm^2^) |  |
| OOS | 987 | Caucasian | 49.6 | 50.3(18.3) | 1.71(0.10) | 80.10(17.72) | 1.03(0.16) | Hologic QDR 4500W |
| KCOS | 2250 | Caucasian | 75.9 | 51.4(13.8) | 1.66(0.08) | 75.16(17.47) | 1.02(0.16) | Hologic QDR 4500W |
| COS | 1547 | Han Chinese | 50.7 | 34.8(13.4) | 1.64(0.08) | 60.27(10.54) | 0.95(0.13) | Hologic QDR 4500W |
| WHI-AA | 712 | African American | 100.0 | 60.9(6.9) | 1.62(0.06) | 83.15(17.72) | 1.05(0.17) | Hologic QDR-2000 |
| WHI-HIS | 409 | Hispanic | 100.0 | 60.7(7.2) | 1.57(0.06) | 73.87(15.62) | 0.97(0.16) | Hologic QDR-2000 |

Notes: Data were presented as mean (SD). Abbreviations: OOS, Omaha osteoporosis study; KCOS, Kansas-city osteoporosis study; COS, China osteoporosis study; WHI-AA, Women’s health initiative African American sample; WHI-HIS, Women’s health initiative Hispanic sample; LS BMD, Lumbar spine bone mineral density. The BMD values of WHI-AA and WHI-HIS samples were measured by DXA using a Hologic QDR densitometer Model 2000 Fan-beam technology (Hologic, Inc.).

**Supplementary Figure 1. Distribution of CpG-SNPs in distinct genomic features.** The potential functional CpG-SNPs were mapped to different elements and compared to the overall distribution of all CpG-SNPs in the human genome.


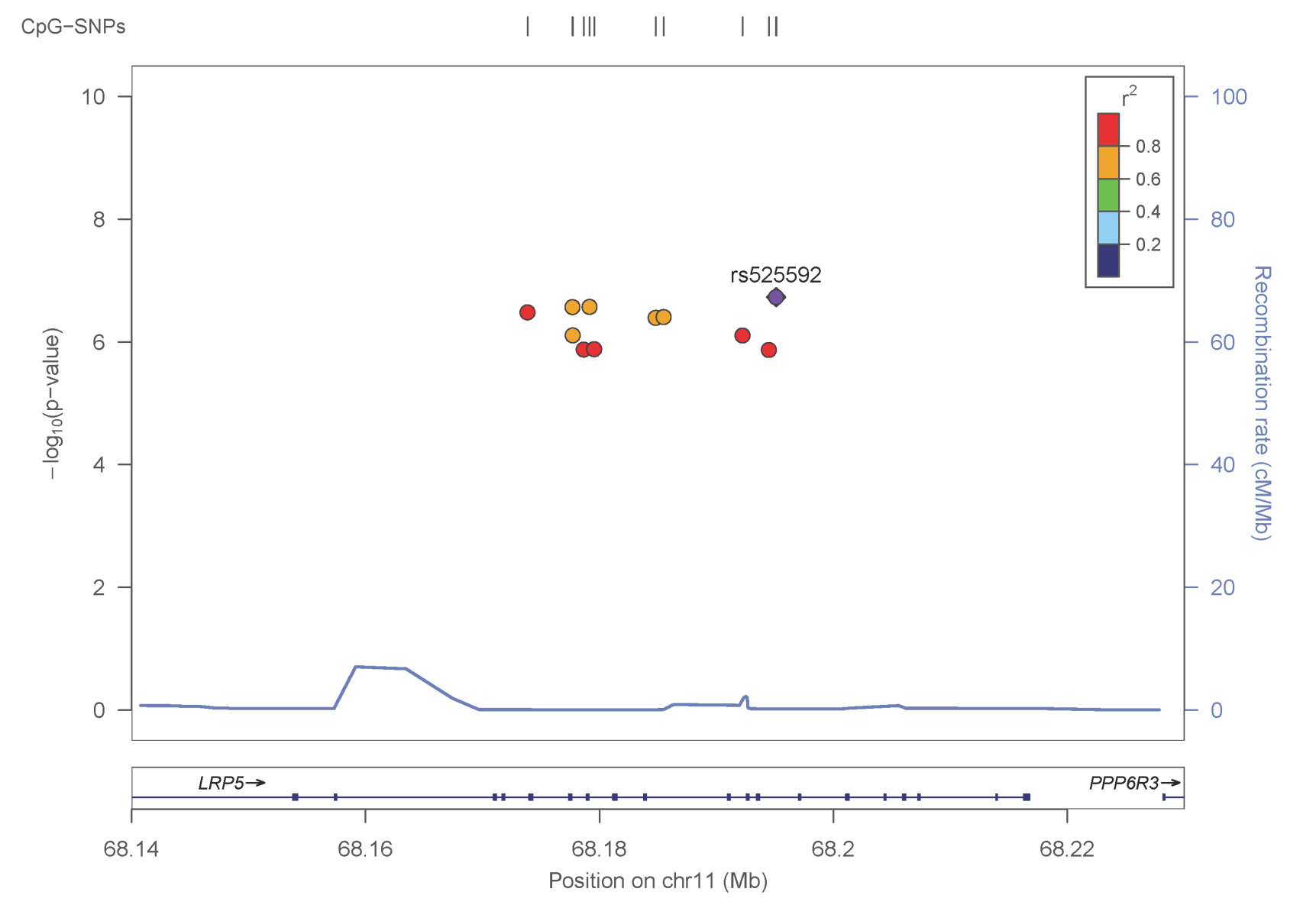


**Supplementary Figure 2**. **A regional association plots of significant/suggestive CpG-SNPs at *LRP5* regions**. Genes and expressed sequence tag (ESTs) within the region are shown in the lower panel, and the unbroken blue line indicates the recombination rate within the region. Each filled circle represents the p-value for one SNP in the meta-analysis, with the central SNP *rs525592* shown in purple and SNPs in the region colored depending on their degree of correlation (r^2^) with *rs525592*.
